# A Phosphenotron Device for Sensoric Spatial Resolution of Phosphenes within the Visual Field Using Non-Invasive Transcranial Alternating Current Stimulation

**DOI:** 10.3390/s24082512

**Published:** 2024-04-14

**Authors:** Faraz Sadrzadeh-Afsharazar, Alexandre Douplik

**Affiliations:** 1 Photonics Group, Department of Physics, Faculty of Science, Toronto Metropolitan University (Formerly Ryerson University), Toronto, ON M5B 2K3, Canada; fsadrzad@torontomu.ca; 2 Institute for Biomedical Engineering, Science and Technology (iBEST), Keenan Research Centre of the Li Ka Shing (LKS) Knowledge Institute, St. Michael Hospital, Toronto, ON M5B 1T8, Canada

**Keywords:** phosphenes, electrical stimulation, visual prosthesis

## Abstract

This study presents phosphenotron, a device for enhancing the sensory spatial resolution of phosphenes in the visual field (VF). The phosphenotron employs a non-invasive transcranial alternating current stimulation (NITACS) to modulate brain activity by applying weak electrical currents to the scalp or face. NITACS’s unique application induces phosphenes, a phenomenon where light is perceived without external stimuli. Unlike previous invasive methods, NITACS offers a non-invasive approach to create these effects. The study focused on assessing the spatial resolution of NITACS-induced phosphenes, crucial for advancements in visual aid technology and neuroscience. Eight participants were subjected to NITACS using a novel electrode arrangement around the eye orbits. Results showed that NITACS could generate spatially defined phosphene patterns in the VF, varying among individuals but consistently appearing within their VF and remaining stable through multiple stimulations. The study established optimal parameters for vibrant phosphene induction without discomfort and identified electrode positions that altered phosphene locations within different VF regions. Receiver Operating characteristics analysis indicated a specificity of 70.7%, sensitivity of 73.9%, and a control trial accuracy of 98.4%. These findings suggest that NITACS is a promising, reliable method for non-invasive visual perception modulation through phosphene generation.

## 1. Background and Introduction

Non-Invasive Transcranial Alternating Current Stimulation (NITACS) is a technique that applies weak electrical currents to the scalp or face to modulate brain activity. NITACS is an increasingly significant tool used to explore the correlation between weak electrical stimulation and the visual phenomenon known as phosphenes [1,2,3,4,5]. NITACS typically employs transcutaneous delivery, varying factors such as electrode positioning, current strength, and stimulation frequency to elicit phosphene experiences [6,7,8,9]. Previous works have studied the electrical stimulation of some areas of the head and the occurrence of phosphenes in the visual field (VF). Yet, the debate over whether phosphenes originate from cortical or retinal stimulation remains open [10,11,12,13]. These studies investigated various aspects of the phosphene experience, including its presence or absence, perception intensity, frequency, and colour [14,15,16,17,18,19,20]. Additionally, NITACS has been probed for its potential to enhance visual perception or cognitive abilities in visually impaired patients through retinal or brain stimulation [21,22,23].

Phosphenes have been observed and studied for many years, and they have been induced through various methods, including electrical stimulation of the visual cortex. NITACS offers a non-invasive approach to induce phosphenes by applying alternating current stimulation to the scalp or face, thereby modulating cortical activity. Understanding the spatial resolution of phosphenes induced by NITACS is crucial for potential applications in vision restoration, visual prosthesis technology, and neuroscientific research.

Research into electrically stimulated phosphenes traces back to 1755 when Charles LeRoy developed the first visual prosthesis exploiting this effect. Shutter and Hortensius (2010) emphasized retinal stimulation’s pivotal role in phosphene perception [13]. Granley and Beyeler [14] created computer models to predict phosphenes’ shape, intensity, and location during invasive retinal stimulation. Bókkon [15] introduced the concept of biophoton bioluminescence as the underlying mechanism for phosphene perception, suggesting electric fields above a certain threshold trigger photon emission, creating perceptions of light. This threshold-dependent nature implies the potential for generating spatially resolved phosphenes by applying electric field gradients above the threshold in specific retina regions, allowing localized light perception. By manipulating the electric field’s spatial characteristics through an electrode array (vectoring), it may be possible to induce spatially resolved phosphenes non-invasively. To our best knowledge, a literature review finds no prior literature on the comprehensive detailed technical design and experimental implementation of a multi-channel, non-invasive phosphene stimulator. Our group is the first to develop a non-invasive platform for exploring phosphene spatial resolution.

Our study sought to discern how phosphenes can be localized within the VF. More specifically, we explored how NITACS can generate spatially resolved phosphenes within the VF, which was prompted by the desire to develop visual prosthetics via phosphene stimulation [24,25,26,27,28]. Our approach involved placing stimulating electrodes around the left and right eye orbits. This study also aimed to investigate the consistency of electrically induced visual percepts (phosphenes) within an individual and across a population, using controlled superficial cutaneous electrical stimulation parameters supplemented by placebo-controlled trials.

Our study encourages further exploration into the origins of phosphenes generated via NITACS when electrodes are relocated across the scalp or face. It also presents a form of stimulation technology that can comfortably and painlessly stimulate multiple differently shaped and located phosphenes within the VF. We implemented this technology in a device we named a “phosphenotron”.

**Figure 1:**
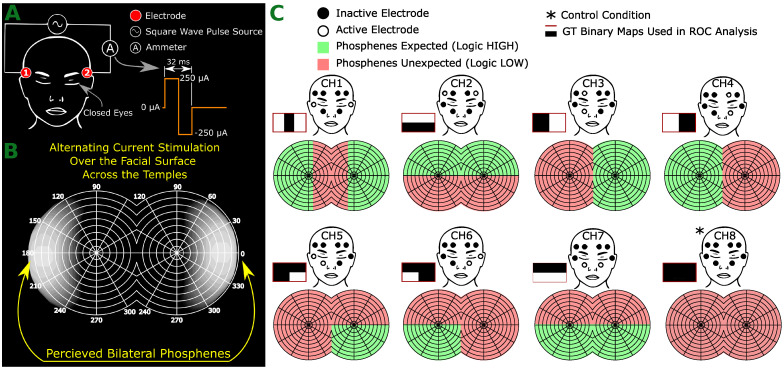
Conceptual overview and hypothesis specifics: (**A**)—pilot study setup with an electrode pair on the temples and a balanced waveform applied. Participants, with closed eyes, reported any phosphenes seen. Electrode positions were varied across facial areas to examine changes in phosphene spatial characteristics. (**B**)—Phosphene observed in the pilot study. (**C**)—Anticipated phosphene locations for each electrode position and ground truth maps for each electrode configuration, aiding in Receiver Operating Characteristic (ROC) analysis.

A pilot study commenced with the primary objective of determining the optimal electrical stimulation parameters. This includes the shape of the waveform, peak current intensity, and electrode size. The goal was to consistently evoke phosphenes without causing skin irritation or discomfort. For this, two visually abled volunteers were equipped with a square pulse generator connected across their temples, which included an ammeter for real-time measurements. Figure 1 displays the pilot study’s experimental setup. After a series of iterative tests, it was deduced that the electrical stimulation parameters represented in Figure 1 consistently evoked phosphenes. It was evident that the frequency of phosphene perception (flashing rate) was identical to the stimulation frequency.

One key observation from this phase was that the appearance of the phosphene was proximate to the location of the stimulation electrodes. This finding led to the hypothetical interpretation that this proximity could remain consistent across varied stimulation configurations. This observation was a significant inspiration for the hypothesis that drove the subsequent study phase.

The study’s hypothesis suggests the possibility of generating spatially encoded phosphenes via electrical stimulation of the facial skin applied around the eye orbits. This is based on the presumption that neurologically active tissues within the visual sensory system, like the retina or optic nerve, closer to the electrodes, experience a stronger electrical field. As a result, these proximal tissues would receive more stimulation than tissues further away.

## 2. Results and Discussion

We elicited visual sensations in eight visually abled subjects (75% males (n = 6), 25% females (n = 2), mean ± SD age: 31.3 ± 14.8). Eight facial skin areas were chosen for each individual to be electrically stimulated in multiple trial events. We hypothesized a concept of spatial perception for the eight tested electrode setups: control (no actual stimulation) and seven other pre-determined facial skin areas. The stimulation was executed one channel at a time. After each trial, participants documented the perceived phosphenes through sketches on a smartboard. A comparison between each localized phosphene drawn on a smartboard and its anticipated pattern (Figure 1) underwent Receiver Operating Characteristic (ROC) analysis using the sensitivity and specificity metrics. This analysis produced four sub-variable figures: true positive, false positive, true negative, and false negative.

**Figure 2:**
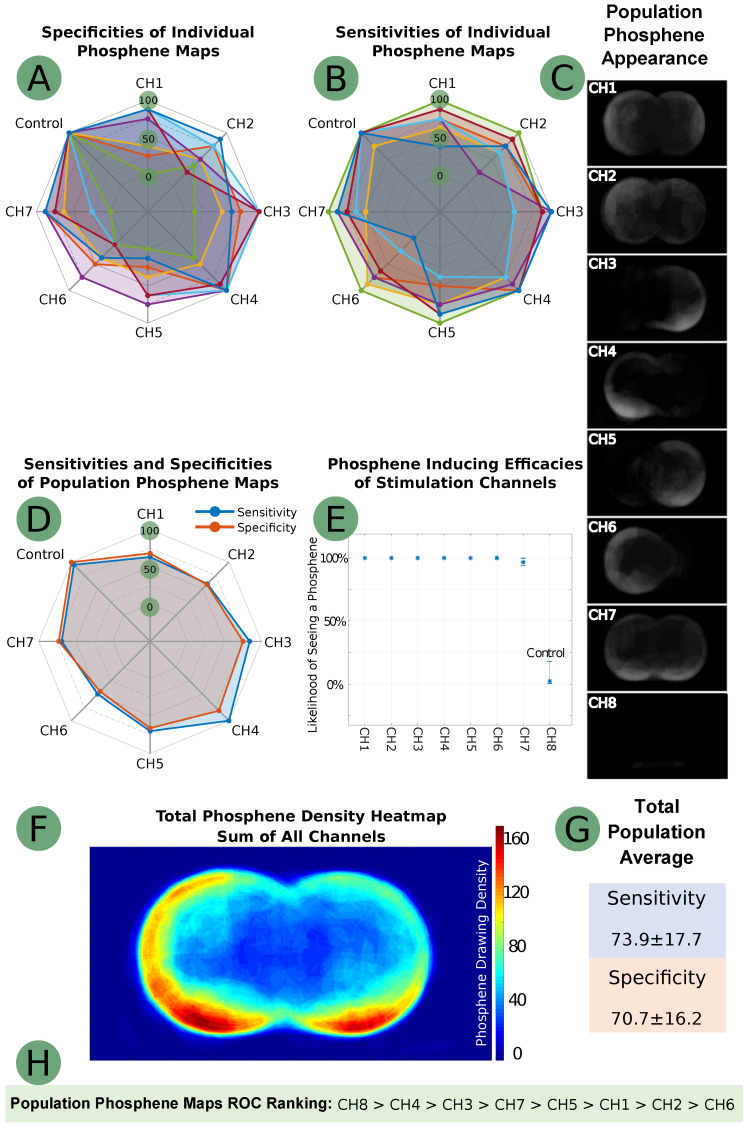
Results data from the NITACS study: (**A**)—individual map specificities; (**B**)—individual map sensitivities; (**C**)—population maps; (**D**)—sensitives and specificities of population maps; (**E**)—efficacy figures for stimulation channels; (**F**)—phosphene density heatmap; (**G**)—average and deviation for population map sensitivities and specificities; (**H**)—ROC analysis rankings of the stimulation channels.

The fidelity of each phosphene map to its hypothesized pattern varied among the study population according to sensitivity and specificity metrics. For channels 1–8, shown in Figure 2G, the proposed electrode setup for channels 1–8 yielded average sensitivity and specificity scores of 73.9% and 70.7%, respectively. The control trial was 98.4% effective in producing no phosphenes.

Regarding channel-based ROC analysis, channels 4 and 3 had the highest ROC scores (excluding control), followed by channels 7 and 5. Channels 3 and 4 showed the highest sensitivity and specificity (84.4% and 75% (CH3), next to the blind control, and 100% and 81.3% (CH4)), and ranked third and second in the ROC ranking. Channel 6 did not demonstrate significant sensitivity and specificity (50% and 45.3%), while the other channels demonstrated a statistically significant ability to encode spatial information. The suboptimal performance of CH6 is difficult to rationalize; it could be due to the high proportion of right-handed individuals in the tested population, where the left half of the VF is less perceptible in terms of spatial resolution and discrimination. Nevertheless, more research is needed to determine the malperformance of CH6. The blind control trial achieved the highest ROC score, suggesting a strong immunity to placebo in our phosphene stimulation method. The ROC rankings are summarized in Figure 2H.

Figure 2A–C details the ROC metrics for all individuals and populations and the renderings of the net summation of the drawings for each stimulation channel. Further testing revealed that electrically stimulated phosphenes do not always occur precisely near the stimulating electrodes, contrary to our initial hypothesis. For example, channels 5 and 6 were found to generate phosphenes in the upper parts of the VF, while the corresponding electrodes were placed on the temples and cheekbones. For the most effective phosphene maps (CH3 and CH4), ROC analysis suggested that phosphenes appear most near the stimulation electrodes (Figure 2C). The ROC ground truth maps were constructed with a crude approach, occasionally expecting that up to half of the VF would perceive phosphenes. Consequently, our hypothesis of “phosphenes manifesting proximal to stimulation electrodes” is defined in a manner that is both crude and broad. The downward orientation of CH3 and CH4 remains open to hypothetical interpretation. The lower impedance of the cheek area (due to higher water content) may have an ohmic and capacitive lensing effect on the volumetric electrical conduction, causing the phosphenes to be more pronounced in the lower part of the VF.

After aggregating all population phosphene maps into a comprehensive phosphene map, we found that phosphenes stimulated by our method tend to appear in the peripheral VF, as depicted in Figure 2F. According to the collected phosphene drawings, the central VF generally lacks phosphenes.

**Figure 3:**
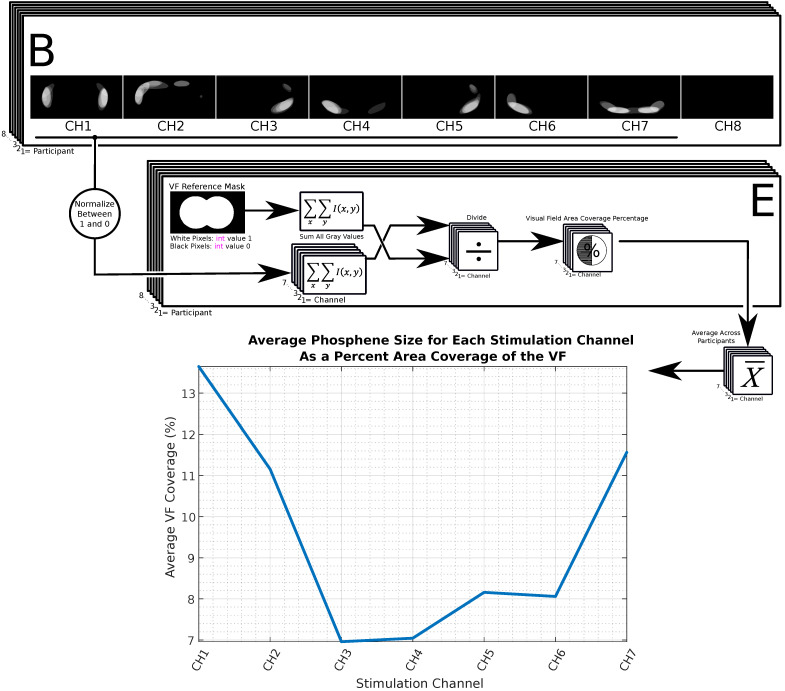
Calculating the percentage of VF coverage for each stimulation channel is key in visual prosthesis. Smaller phosphenes allow for higher-resolution spatial perception to the user.

The process described in Figure 3 involves a post-processing step to calculate the sizes of phosphene blobs regarding VF area coverage. It involves normalizing the grey values of individual phosphene maps between 0 and 1. Then, a special binary mask is conceived to highlight the area enclosed by the outer contours of the perimetry target. This mask represents the maximum hypothetically possible phosphene size (which covers the entire VF). Ratios are obtained by dividing the sum of grey values in each phosphene map by the sum of grey values in the perimetry mask. These ratios are labelled channel-specifically and averaged across participants, expressed as percentages. Channels 3 and 4 showed the highest spatial concentration, while others had larger phosphenes. Smaller, more distinct phosphenes are preferred as they provide higher-resolution spatial information, essential for an ideal visual communication tool offering ample bandwidth to the user. This analysis implies that a visual prosthesis using this technology would work best with smaller phosphenes for clearer distinction due to their localized nature.

Our stimulation technique was also shown to be highly efficient in inducing phosphenes. Across all non-control channels, the average effectiveness in producing phosphenes was 99% (excluding control). Figure 2E provides a detailed breakdown of the efficacy per channel for phosphene stimulation.

These results open up possibilities for visual guidance prosthetic technologies. Our results suggest that NITACS can be used as a non-invasive tool for investigating a causal link between the location of the facial stimulation site and the localization of the phosphenes within the field of vision. To ensure the experiment’s safety, measures were taken based on preceding transcranial alternating current stimulation literature. The stimulation current was limited to under 500 μA, half of what is commonly permitted [1] and FDA approved for contemporary brain stimulation devices [6]. The active stimulation duration did not exceed 90 s, significantly less than the 30 min cited by Carvalho, Leite, and Fregni [1]. Additionally, a large electrode contact area and Ten20 paste were used to distribute the current evenly and minimize discomfort and injury risk.

We identified two subgroups of four individuals each, with combined sensitivity and specificity scores of 114 and 198 (out of 200%), possibly indicating an inherent propensity for phosphene perception. Conditionally, we referred to these groups as the “strong phosphene responsive group” and “weak phosphene responsive group”, or simply the strong and weak groups. Although we cannot precisely explain why the current stimulation setup and electrode placements resulted in less than 100% spatially resolved true positives (according to Figure 2), it may depend on an individual’s inherent ability to perceive phosphenes. This is supported by the sensitivity and specificity of 79.7 ± 23.3% and 72.7 ± 28.3% for the strong group versus 68.1 ± 20.8% and 68.7 ± 31.7% for the weak group, although both the strong and weak groups showed statistically significant results.

We cannot fully explain why the central VF generally lacks phosphenes, as the electrodes facilitate the current passing across the eye orbit areas. Our next phase of the study will focus on this phenomenon.

The study’s findings provide insightful data about the spatial resolution of phosphenes induced by non-invasive transcutaneous electrical stimulation. Ref. [29] focuses on significant outcomes of the bespoke study above, such as the optimal stimulation parameters and the ROC performance figures of the top-performing stimulation channels. This paper delves into all stimulation channels’ methodologies and ROC performance figures. This paper also dives deeper into the current methodologies and past research on the topic.

The results’ interpretation should consider the limited sample size, which might impact their broad applicability. Larger human studies are needed to understand better the relationship between skin stimulation sites and the spatial properties of phosphenes in the VF.

## 3. Limitations and Future Study

The unique spatially resolved perception of NITACS phosphenes inspires the potential for understanding fundamental phosphene perception mechanisms and their real-world applications. Individual differences in perception highlight the need for further research, including enhancing phosphene response through training and optimizing stimulation for integrated visualizations (drawing shapes, lines, and text). Ongoing work focuses on a prototype combining a phosphene stimulator with camera and machine vision to target central VF phosphenes (hopefully achievable), offering advanced stimulation options (e.g., higher electrode count, waveform shaping capability, arbitrary electrode pairing, and simultaneous stimulation of many electrode pairs).

The study employed equity, diversity, and inclusion in participant recruitment, randomly selecting across genders and sexes within the pool’s size constraints. Future research should broaden recruitment to include broader and more diverse gender identities, sex groups, ages, and racial demographics.

Future research should especially prioritize enlarging human study sample sizes to deepen understanding of phenomena and applications, building on the initial proof of principle. Complementary finite element and phantom studies will enhance overall insight.

Human studies should eventually target the visually impaired if the technology proves useful for prosthetics, pending further proof-of-concept studies before clinical research. As it stands, this research may not yet be generalized to clinical populations.

The current study focuses on participants’ subjective experiences while not focusing on the objective study of phosphenes. Future endeavours may delve into the neuroscientific study of phosphenes, scanning the brain (fNIRS, fMRI, EEG, MEG, etc…) during stimulation.

A safety and efficacy study is needed with a focus on the long-term side effects of this method. Electromagnetic dosimetry calculations and simulations in the low frequencies may add insight into the efficacy and safety of the long-term use of the phosphenotron.

Lastly, the effects of brain laterality and handedness will be investigated in relation to the spatial characteristics of phosphenes.

## 4. Materials and Methods

### 4.1. Feasibility Study on Visually Abled Human Volunteers

**Figure 4:**
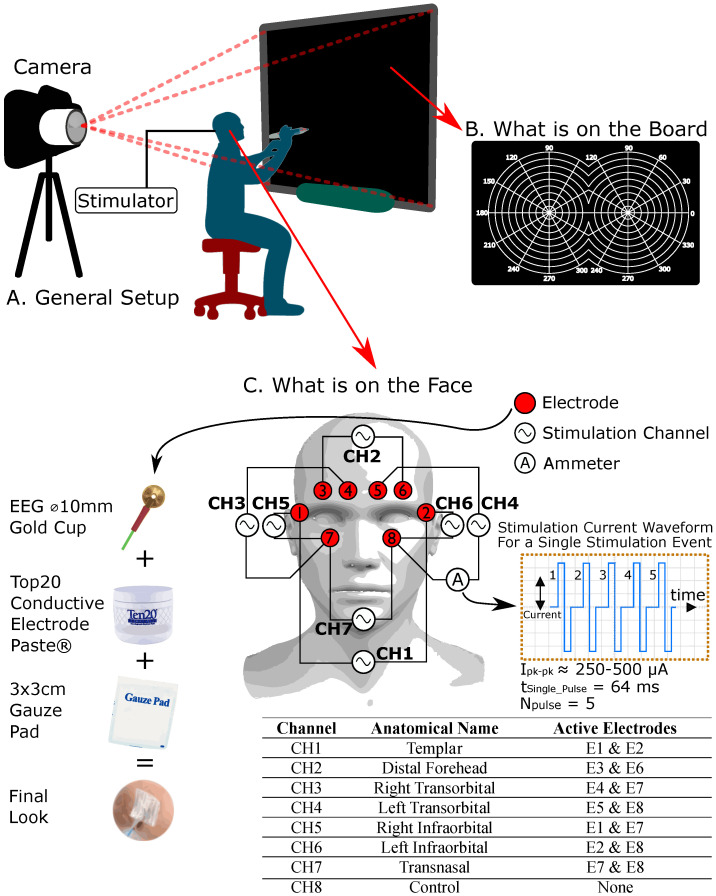
The experimental setup of the NITACS study. The electrode placement and stimulation waveform configuration for the human trials. This figure also outlines the anatomical and electrode wiring detail associated with each stimulation channel.

In this phase, each participant was equipped with the electrode dressing depicted in Figure 3. The dressing included eight EEG gold cup electrodes arranged around the orbital sockets. The stimulation channels (CH1, 2, 3, …, 7) were connected to electrodes numbered 1 through 8. A control channel (CH8) was also created for use during control trials. There was no stimulation waveform across any electrode during a control trial. Each stimulation channel was assigned an anatomical name corresponding to the location of its respective electrode pair on the face. The anatomical names for each stimulation channel are provided in Figure 4.

### 4.2. Experimental Parameters

The feasibility study, conducted with eight visually abled volunteers, aimed to map the location and shape of phosphenes across a healthy population, laying the groundwork for future research models. All methods were carried out in accordance with relevant guidelines and regulations, as outlined in the Research Ethics Board (REB) protocol. The recruitment procedure included placing posters on bulletin boards around the hosting institution and inviting passersby to volunteer for the study. The invitees would be screened using a questionnaire to ensure that the volunteers are visually abled, between the ages of 20–50 years, and have no history of migraines, panic attacks, and/or agoraphobia. This measure was taken to exclude vulnerable individuals in the starting phase of the research, therefore minimizing risk.

The research protocol was reviewed and approved by the Ryerson University (now Toronto Metropolitan University) REB committee (REB file number: 2019-324). Informed consent was obtained from all subjects before participating. Each participant was led into a dimly lit room and sat on a stool 50 cm away from a smartboard (Figure 4A), which displayed an ophthalmological binocular perimetry target (Figure 4B). This target phenomenologically represents the combined VF of both eyes. As shown in Figure 4A, an off-axis camera was set up to capture the participants’ drawings on the smart board without being obstructed by the participant’s body. Figure 4C illustrates the participant receiving the EEG gold cup electrodes, which were secured with Ten20 electrode paste and further reinforced with a 3 × 3 cm gauze pad. In the initial step, the phosphene stimulation threshold was determined for each participant by gradually increasing the stimulation current intensity until the participant reported seeing a phosphene. The stimulation intensity was kept below 500 µA for safety [1,30], and once phosphenes were detected, the intensity was held constant for the remainder of the experiment. We noted no fading effects requiring increased current, even after extended stimulation periods (15–20 min). After this step, data collection began. Stimulation sites, called stimulation and control channels (CH1, 2, 3, …, 8), were activated in a pseudo-randomized sequence. A MATLAB script created this sequence, ensuring each channel was stimulated eight times, resulting in 64 stimulation events. Only two electrodes were active during each event, and the participant was asked to sketch the perceived phosphene shape on the smartboard (Figure 4A,B). These sketches were collected as phosphene renderings and used for further data analysis. Due to pseudo-randomization, each photograph was traced and linked to its associated channel, making the stimulation sequence appear random to the participant. Upon completion of all stimulation trials, the electrodes were removed, and the participant’s skin was cleaned.

### 4.3. Stimulator Technology

**Figure 5:**
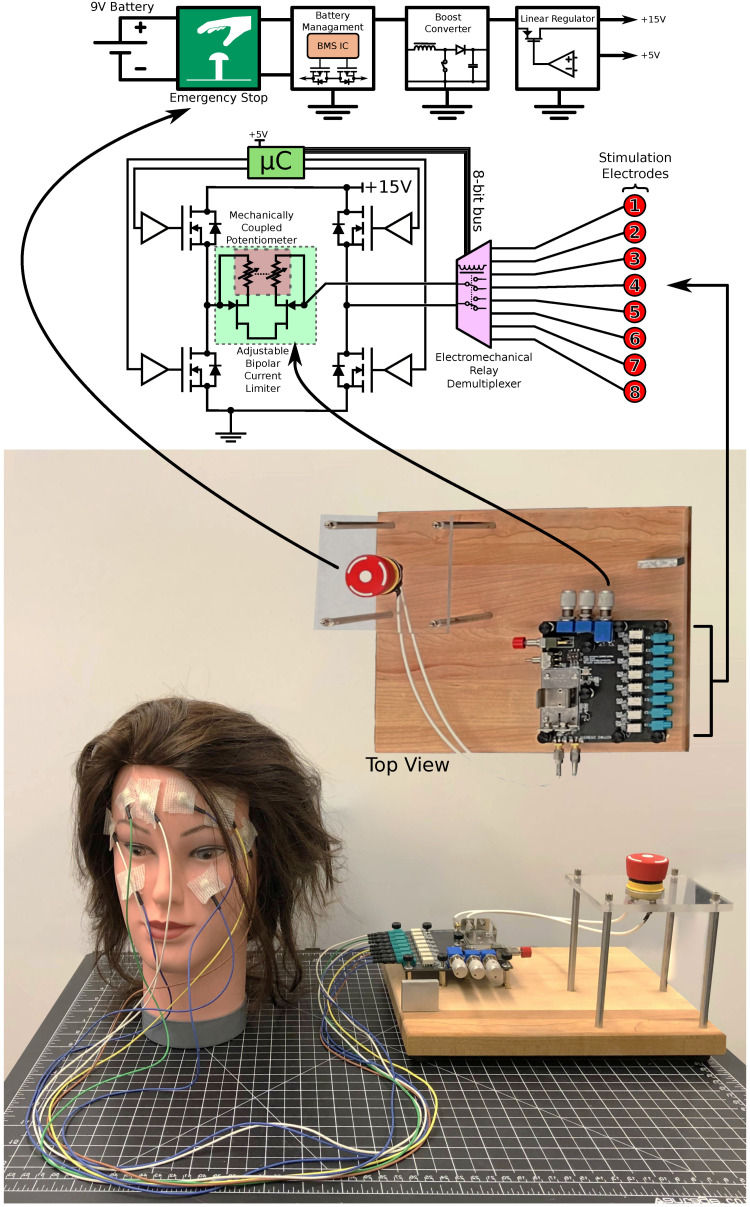
The circuit architecture and the experimental stimulator setup. The image depicts both the power path and the stimulation path of the stimulator.

The stimulator, tailored for human studies, aimed to understand how face stimulation locations correlate with phosphenes in the VF. Its electronics and use case, detailed in Figure 5, include a simplified electronics process flow and a lumped electrical model for eight channels. This prototype was battery-powered, using a 9 V battery and designed as a desktop device. It featured an emergency button (as a power button), a boost converter, and linear regulation) to achieve a low noise 15 V voltage rail. The bi-phasic waveform was generated using a dual gate driver (2EDN8524FXTMA1, Infineon Technologies, Germany). This setup and eight double-pole-double-throw electromechanical relays allowed stimulation waveform demultiplexing across multiple electrode pairs. A depletion-mode field effect transistor pair (LND250, Microchip Inc., USA) in a common-drained configuration was used for current limiting during stimulation, utilizing their saturation regions. This was achieved by connecting a double-ganged rheostat in series with the source terminals of the transistors and linking the gate. Placing this circuit in series with the stimulation path allows the continuous adjustment of the current limit (50–500 μA). A microcontroller is used to sequence a series of actions that would lead to the creation of the stimulation waveform. Two other potentiometers are used to collect user input to set the stimulation frequency and pulse width. The device could not measure the threshold current, but was designed to maintain a set maximum current determined by the position of the double-ganged potentiometer.

### 4.4. Data Post-Processing

**Figure 6:**
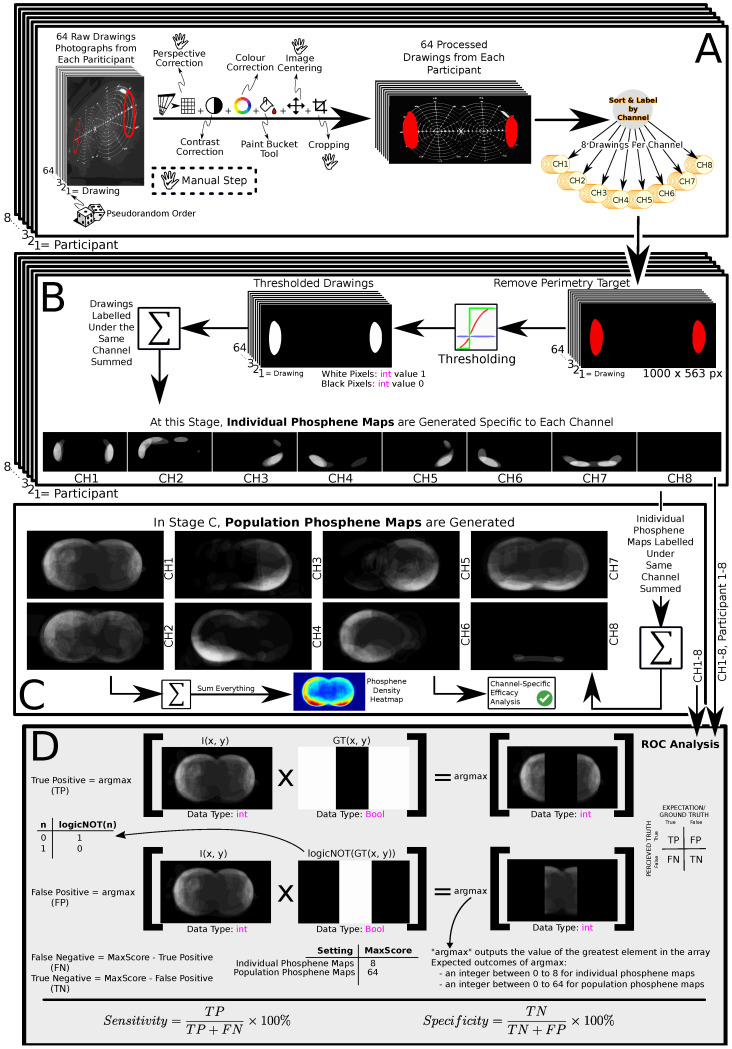
The data processing workflow: (**A**)—pre-processing drawings into labelled drawings; (**B**)—generating individual maps; (**C**)—generating population maps, computing the phosphene density heatmap, and stimulation efficacy analysis; (**D**)—ROC analysis [31].

The main metric used to assess the performance of each channel is the Receiver Operating Characteristic (ROC) metrics of sensitivity and specificity. Sensitivity (in our context) measures the ability to display phosphenes in the channel-specific expected region of the VF. Specificity measures the ability to keep phosphates out of the channel-specific expectation of phosphene locations. ROC analysis uses sub-metrics: True Positive (TP), True Negative (TN), False Positive (FP), and False Negative (FN), to evaluate outcomes. These metrics arise from comparing actual (ground truth) and predicted (perceived truth) outcomes. The relationships among these sub-metrics are detailed in Figure 6D. The TP sub-metric is computed using the ground truth masks (where phosphenes are expected) described earlier. The negative images of the truth masks (where phosphenes are not expected) are used to compute the FP sub-metric. Knowing the maximum possible ROC sub-metric score (different between population and individual map categories), sub-metrics of FN and TN are computed, given that by definition, FN and TN are 100% complements of TP and FP, respectively [31].

Each participant’s raw phosphene drawings were transformed post-trial into individual and population phosphene maps for ROC analysis (Figure 6D) [31]. The initial data were registered via a smartboard. The phosphene drawings were then isolated from their perimetry target background and thresholded to create a binary mask representing the phosphene bodies as perceived by the participants (Figure 6A). Individual phosphene maps were generated by adding the drawings under the same stimulation channel, resulting in eight individual phosphene maps for each participant. ROC analysis was applied to each phosphene map (Figure 6B,D).

The binary masks proposed in Figure 1C served as the ground truth in the ROC analysis. They represent the anticipated spatial locations of phosphenes associated with each electrode (Figure 6D).

Population phosphene maps were produced by adding individual phosphene maps of the same stimulation channel (Figure 6C). ROC analysis was also applied to these population phosphene maps.

An additional step was performed, in which each stimulation channel was assessed for its ability to generate phosphenes. These figures (referred to as the phosphene stimulation efficacy analysis) were computed as a percentage of channel-specific trials in which phosphene perception was reported. This operation aimed to see whether different stimulation channels differ in their ability to induce phosphenes at given stimulation currents (Figure 6C).

Phosphene drawings’ sizes, shown as a percentage of the total visual field (VF) area, were evaluated based on their relative average size per channel, as depicted in Figure 3.

Finally, a complete summation of all phosphene drawings was computed to ascertain the spatial orientation of the phosphenes stimulated on the face. The steps involved in Figure 6 were executed in MATLAB (Version R2020a, MathWorks, USA).

## 5. Conclusions

This study presents phosphneotron, a novel cutaneously mounted device for creating spatially resolved, non-invasive, electrically stimulated phosphenes in the VF. We collected and analyzed data to develop individual and collective population phosphene maps, which helped to confirm the reproducibility, population consistency, and geometric characteristics of the perceived phosphenes. Most importantly, the phosphneotron could choose the location of the facial stimulation site, hence controlling the spatially resolved properties of phosphenes, such as their size, location, and intensity. Such a device would open many possibilities for studying NITACS.

The placebo-controlled trials had a phosphene accuracy rate of 98.4 ± 1.5%. Moreover, our results showed that phosphenes were generally absent in the central VF, given the current electrode locations and stimulation settings.

This study can leverage the cost-effective development of visual prostheses that operate on the non-invasive stimulation of facial skin. The ability to move phosphenes in the VF expands the sensory bandwidth of information transfer to a user, thus enabling the communication of concepts such as direction, time, and spatial proximity. While the previous literature [29] focuses on human studies, this work focuses on the stimulator design, as well as conducting a feasibility study exploring potential applicability of our NITACS platform in visual prosthesis technologies.

## Data Availability

Data from the experiment can be requested from the corresponding author via email at douplik@torontomu.ca.
